# Diagnosis on Ultrasound Images for Developmental Dysplasia of the Hip with a Deep Learning-Based Model Focusing on Signal Heterogeneity in the Bone Region

**DOI:** 10.3390/diagnostics15040403

**Published:** 2025-02-07

**Authors:** Hirokazu Shimizu, Ken Enda, Hidenori Koyano, Takuya Ogawa, Daisuke Takahashi, Shinya Tanaka, Norimasa Iwasaki, Tomohiro Shimizu

**Affiliations:** 1 Department of Orthopaedic Surgery, Faculty of Medicine and Graduate School of Medicine, Hokkaido University, Sapporo 060-8638, Japan; rottefella.nnn@gmail.com (H.S.); tkyoztf@yahoo.co.jp (T.O.); rainbow-quest@pop02.odn.ne.jp (D.T.); niwasaki@med.hokudai.ac.jp (N.I.); 2 Department of Cancer Pathology, Faculty of Medicine, Hokkaido University, Sapporo 060-8638, Japan; buhibuhidog@gmail.com (K.E.); tanaka@med.hokudai.ac.jp (S.T.); 3 Department of Medical Physics, Graduate School of Medicine, Hokkaido University, Sapporo 060-8638, Japan; koyano@med.hokudai.ac.jp; 4 WPI-ICReDD (Institute for Chemical Reaction Design and Discovery), Hokkaido University, Sapporo 001-0021, Japan

**Keywords:** quality assessment, ultrasound images, deep learning, signal heterogeneity, developmental dysplasia of the hip

## Abstract

**Background:** Developmental dysplasia of the hip (DDH) is a prevalent issue in infants, with ultrasound crucial for early detection. Existing automatic diagnostic models lack precision due to noise, but 3D technology may enhance it. This study aimed to create and assess a deep-learning-based model for automated DDH diagnosis by using 3D transformation technology on two-dimensional ultrasound images. **Methods:** A retrospective study of 417 infants at risk of DDH used ultrasound images, combining convolutional neural networks and image processing. The images were analyzed using algorithms such as HigherHRNet-W48. The approach included apex point estimation, signal heterogeneity analysis of ilium, which focused on the bony area with high intensity and evaluate ilium rotation, alpha angle creation, and the establishment of a comprehensive method for DDH diagnosis. **Results:** Key findings include: (1) Superior accuracy in apex point estimation by the HigherHRNet-W48 model, even better than orthopedic residents. (2) Thorough quality assessments of ultrasound images, leading to qualified and disqualified categories, with qualified images displaying notably lower error rates. (3) The AUC of the model for DDH detection in the qualifying images was 0.92, exceeding the diagnostic accuracy of the resident, indicating the diagnostic capability of the tool. **Conclusions:** The study developed a deep-learning-based model for DDH detection in infants, melding 3D technology with deep learning to address challenges like noise and rotation in ultrasound images. The study’s innovation demonstrated a comparative accuracy to specialized evaluations, even with non-specialist images, highlighting its potential to assist novice sonographers and enhance diagnostic precision.

## 1. Introduction

The utilization of ultrasound imaging is highly endorsed for the early detection of developmental dysplasia of the hip (DDH) in at-risk infants, primarily due to its safety attributes, including the absence of radiation, non-invasiveness, and the possibility of real-time data acquisition in various settings [1,2,3]. This diagnostic approach is particularly suitable for infants aged 0 to 6 months, as early detection and intervention can help prevent the onset of early adult osteoarthritis [4]. A global trend is emerging towards the establishment of comprehensive imaging-based screening systems for DDH, given its prevalence as one of the most common congenital musculoskeletal abnormalities affecting infants [5]. Consequently, the demand for clinically applicable systems that can efficiently analyze ultrasound images is gradually increasing.

To date, the previous literature studied automated models on infantile images for making automatic diagnoses for DDH with a sufficient value of the area under the curve ranging from 0.89 to 0.97 [6,7,8,9,10,11,12,13]. While existing studies on ultrasound images have primarily concentrated on segmenting essential structures such as the ilium, apex point, and acetabular roof [8,13], they have often overlooked the inherent challenges present in clinical settings. Artifacts, most notably the reverberations around bone, can hinder the accurate evaluation of ultrasound images in practice [14,15,16,17]. Thus, the previous models can face difficulties in analyzing images with artifacts since they did not have an algorithm for noise or artifact reduction. Currently, the following quality assessments are required for analyzing ultrasound images: interference with similar tissues, noise, such as reverberation, and the rotation of the subjects [18].

To attenuate noise and authenticate the quality of ultrasound images, the implementation of 3D technology has been recommended [19,20,21]. This technique likely entails the examination of signal intensity in 2D images, followed by their transformation into a 3D map, effectively reducing the impact of noise. By converting 2D ultrasound images into a 3D format, we have the potential to enhance the images’ precision and clarity, thus improving diagnostic accuracy. The hypothesis of this study was that automated DDH diagnosis could be facilitated by scrutinizing signal inconsistencies within the bone region of 2D ultrasound images.

In our facility, orthopedic residents who are novice sonographers have investigated the ultrasound examinations for infants at risk of DDH, subsequently validated by pediatric orthopedic specialists. This meant that large parts of the recorded ultrasound images for detecting DDH were performed by novice sonographers, containing disqualified images. To address the above hypothesis, the objective of this study was to develop and evaluate the diagnostic performance of a deep learning-based model on infantile ultrasound images for the diagnosis of DDH with an algorithm of noise reduction and the automatic exclusion of disqualified images according to the clinical standard protocols. Since, to the best of our knowledge, there have been no reports describing algorithms for noise reduction by analyzing signal inconsistency in 2D ultrasound images, it has the potential to solve an issue in widely used 2D ultrasound examinations.

## 2. Materials and Methods

### 2.1. Ethics Statement

Our study was performed in accordance with the relevant guidelines of Hokkaido University Hospital and was approved by its Research Ethics Review Committee. The research protocol for human samples used in this study was approved by the Research Ethics Review Committee of Hokkaido University Hospital (approval ID: 019-0022). Informed consent for using samples in our research was obtained from all participants.

### 2.2. Participants and Data Collection

**Figure 1:**
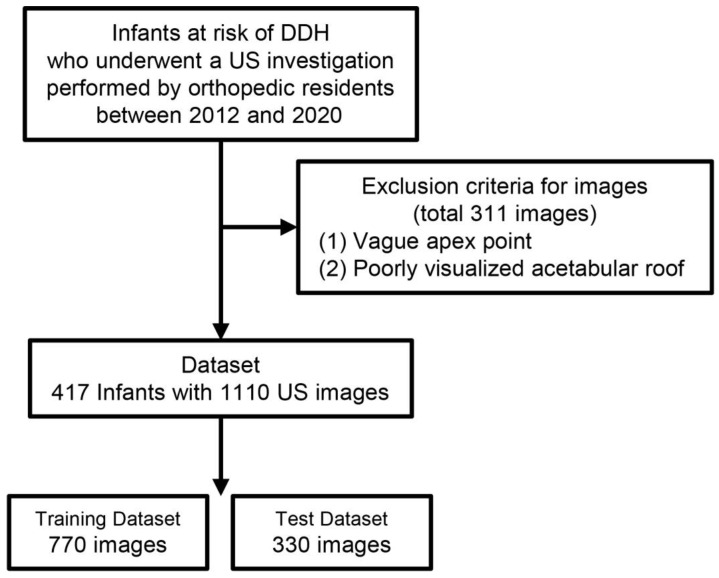
Flow diagram representing the structure and progression of the study.

We enlisted infants at risk of DDH who had visited our hospital’s orthopedic department between 2012 and 2020 and undergone ultrasound examination [22]. Figure 1 presents the flow chart of the study. Infants were often investigated by several orthopedic residents; in some cases, only the ipsilateral side was investigated. The original dataset consisted of 1421 images, in which one image per joint was recorded by the same examiner. We excluded cases based on the following criteria: (1) ambiguity of the apex point structure, and (2) poor visualization of the acetabular roof. Our study encompassed a total of 417 infants (median months (interquartile range), 2 (1–4)) with 1100 ultrasound images, and the demographic data are presented in Table 1. A total of 52.0% (217/417) of the enrolled infants had undergone ultrasound examination twice or more. 

The apex point, which is defined as the point where a line drawn from the inferior end of the ilium meets the ilium, was annotated by two pediatric orthopedic specialists with more than 15 years’ experience. DDH was defined if the alpha angle, which is formed by the acetabular roof to the vertical cortex of the ilium and thus reflects the depth of the bony acetabular roof, measured by the surgeons was less than 60 degrees [23]. Following the clinical protocol, which states that a standard plane should have a vertical ilium, qualifying images were defined as those where the angle of the produced ilium line ranged from 87 to 93 degrees. Deep learning models and orthopedic residents performed the measurements, and their accuracies were subsequently compared in the test dataset.

### 2.3. Framework of Proposed Deep Learning-Based Models

**Figure 2:**
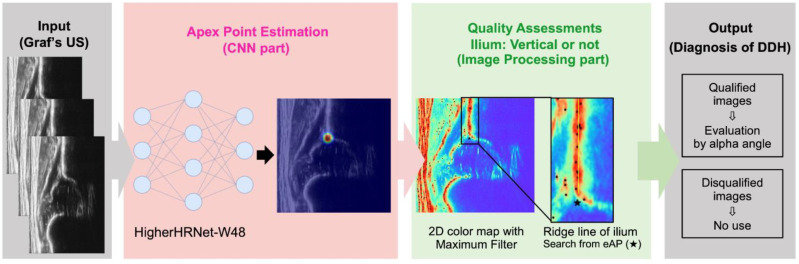
Diagram of the proposed model. Images were analyzed via CNN to determine the apex point, thereby filtering similar tissue. Following this, signal heterogeneity in the bone region around the estimated point (Black star) was examined, ridges on the bone were detected, the ilium’s ridge line was outlined, and a quality assessment was conducted. Only non-rotated, qualified images were used for diagnosis.

Figure 2 provides an overview of the system. For model development, the system integrated separate algorithms: convolutional neural network (CNN) and image processing.

#### 2.3.1. Apex Point Estimation

**Figure 3:**
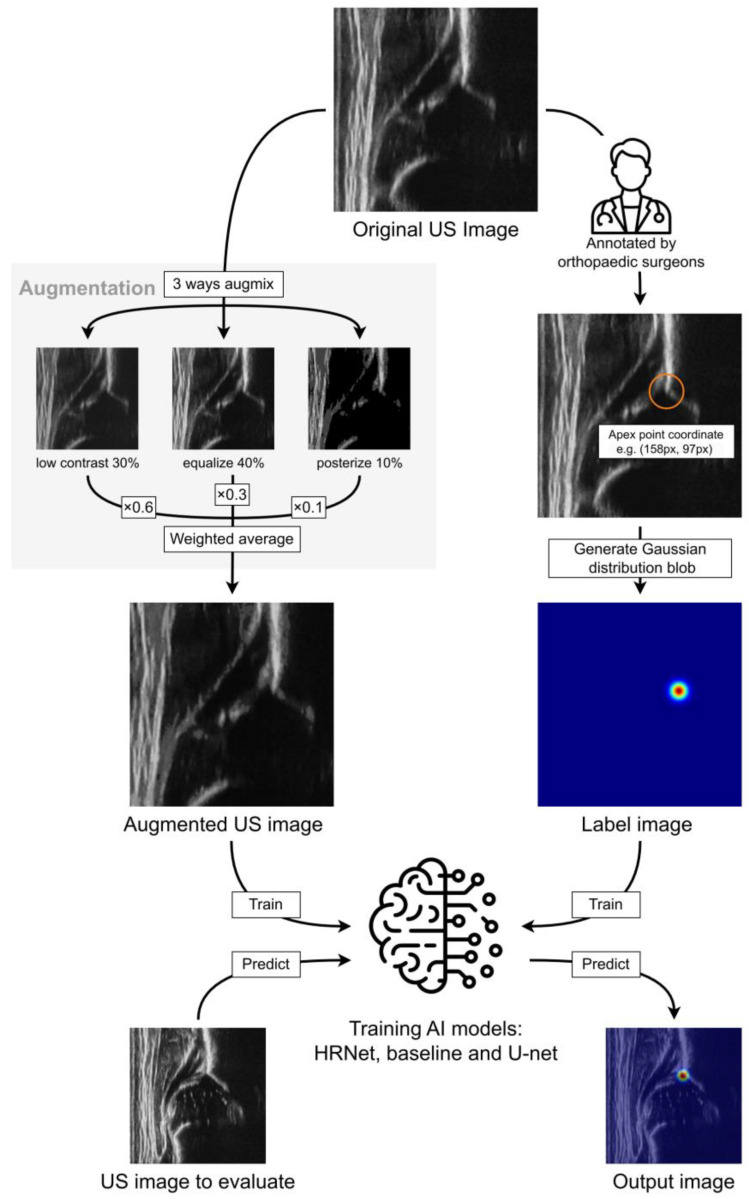
Composition of the Higher HRNet-W48 system for apex point estimation. Inputs are randomly resized (0.875–1.2), cropped at 224px, and augmented through seven processes, with three random effects applied. Processed images are combined in a ratio determined by the Dirichlet distribution, with corresponding training data scaled and shifted before conversion. Numerical values shown are examples from the online random augmentation pipeline.

For data preprocessing, the raw image data were resized into 256 × 256 pixels and converted into PNG files. In this step, a system employing convolutional neural networks can learn and extract a distinctive point, namely, the apex point. For input data, we trained several models, including HigherHRNet-W48 [24]. The specific training flow in this component is detailed in Figure 3.

Each image was randomly resized between 0.875 and 1.2, and cropped at a random position to 224 pixels, followed by image effect augmentation. The augmentation comprised a combination of seven processes, as follows:

1. Equalizing the histogram of each color channel (applied with 50% probability)
2. Reducing the number of bits for each color channel (uniformly sampled between 2 and 4 bits)
3. Inverting pixel color (threshold uniformly sampled from 156 to 256)
4. Increasing contrast (multiplier uniformly sampled from 1.0 to 4.0)
5. Reducing contrast (multiplier uniformly sampled from 0.2 to 1.0)
6. Increasing brightness (multiplier uniformly sampled from 1.0 to 3.0)
7. Reducing brightness (multiplier uniformly sampled from 0.4 to 1.0)

Three randomly chosen effects from the seven listed above were applied to the image with strength levels uniformly sampled from a range of 0.2–0.8. These processed images were then combined using weights generated from a Dirichlet distribution (α = 1), a technique based on AugMix [25]. The training data corresponding to each image were scaled and moved similarly before being converted into a map image or a rectangle.

To gauge the superiority of HigherHRNet-W48, other CNN models were also trained and compared, including HRNet, Baseline model, and U-Net (with encoders as VGG11, 13, 16, 19) [25,26,27,28,29,30,31,32]. All the models were trained using binary cross-entropy, and the accuracy of the estimated apex points (eAP) was compared.

#### 2.3.2. Quality Assessment Through Signal Heterogeneity Analysis

In this component, image processing techniques incorporating OpenCV were applied. We established a region of interest (ROI) based on the eAP. Based on the mean distance of 4.068 pixels between the ground truth and the eAP, this ROI has a 10-pixel width with the midpoint set as the X coordinate of the eAP. The ROI has no limitation on the upper side while the bottom line is 5 pixels away from the Y coordinate of the eAP, assuming that certain ratios of the ROIs could contain the ground truth of the apex point.

**Figure 4:**
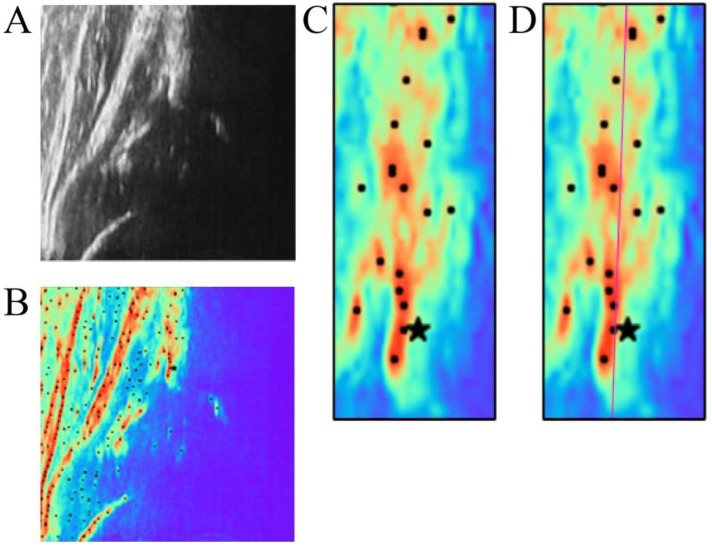
Illustration of signal heterogeneity in the bone region: (**A**) Grayscale images; (**B**) 2D color map and local maximum points; (**C**) Signal heterogeneity in bone region; (**D**) Ridge line (pink) on the bone region.

**Figure 5:**
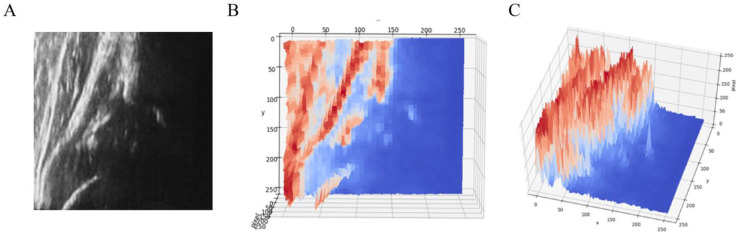
2D image transferred into 3D map images. (**A**) Grayscale. (**B**) 3D color map. (**C**) 3D color map from another angle.

To detect signal heterogeneity in the ROI, we identified points of local maximum pixel value in the ultrasound image, which was converted to a 2D color map from grayscale for better visualization, as shown in Figure 4. These local maximum points were obtained by filtering with maximum neighboring pixels and masking the filtered image with the original image. Figure 5 demonstrates how the 2D images were also transferred to a 3D map.

**Figure 6:**
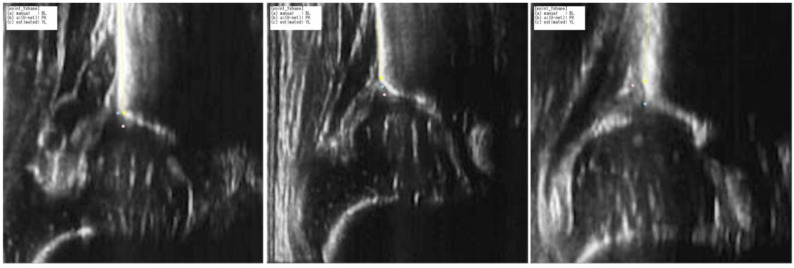
Representative images showing the apex point annotated by the specialist (ground truth, blue), HigherHR-W48 (estimated apex point: eAP, pink), and the point that created the selected local maximum points (yellow).

Subsequently, the ridge line of the ilium was delineated based on the least mean approximation of the selected local maximum points. The representative images of the ridge line, the points creating the selected local maximum points, the ground truth, and the eAP are shown in Figure 6.

The quality assessment was then performed in accordance with this ridge line. Since there have been no reports of strict criteria for the acceptable range of the iliac angle, we set it at 87–93° in this study.

#### 2.3.3. Generation of the Alpha Angle

This process is detailed in Figure 6. The point indicating the edge of the acetabular roof is defined as the lowest Y-coordinate within the rectangle. Based on this point, we selected the maximum points encompassed from the horizontal line to the diagonal line at a 45-degree angle. Both of these lines originate from the point indicating the edge of the acetabular roof. The ridge line of the acetabular roof is derived from the least mean approximation of the selected local maximum points. These two ridge lines form the alpha angle.

### 2.4. Implementation Details

In the first step involving the CNN, the experiments were conducted on a computer equipped with a CPU^®^ Ryzen™ 9 5950X@3.4GHz, 64 GB of RAM (Advanced Micro Devices, Santa Clara, CA, USA), and a GPU NVIDIA^®^ GeForce RTX™ 3090 (NVIDIA, Santa Clara, CA, USA). In the second step, utilizing image processing, the experiments were performed on a computer featuring an Intel^®^ Xeon^®^ CPU ES-2630 v3 @2.40GHz 2.40 GHz processor and 32.0 GB of RAM.

### 2.5. Statistics Analysis

The student’s *t*-test was used to compare the distance from the ground truth between the fitted model and the orthopedic residents, and the inter-rater reliability and intra-rater reliability between qualified and disqualified images. The performance of the fitted model was then analyzed with the receiver operating characteristic (ROC) curve (AUC). Diagnostic sensitivity and specificity of the fitted model and the orthopedic residents were investigated with a cut-off value set at an α angle of 60° or less. All statistical analyses were performed using SPSS Statistics version 23.0 (IBM Corporation, Armonk, NY, USA) with a significance level of 0.05.

## 3. Results

### 3.1. Step1: Accuracy of the Estimated Apex Point

**Figure 7:**
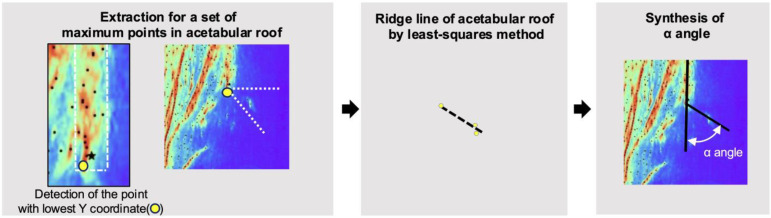
Production of the ridge line and alpha angle. Flow chart showing the production of the ridge line of acetabular roof. The maximum point with lowest Y coordinate in the rectangle was detected. From that point, a set of maximum points of acetabular roof was searched; area was determined by horizontal line and diagonal line with 45 degrees. ROI: region of interest.

Table 2 displays the accuracy of the eAP as determined by various CNN models. In this context, “distance” refers to the pixel difference between the ground truth and estimated apex point. HigherHRNet-W48 had a distance (pixel) between the ground truth and the estimated apex points with a mean (± standard error) of 4.068 (±0.088). Among the most representative three models (The HigherHRNet-W48, U-net [BGG16], and Baseline 101), HigherHRNet-W48 showed the smallest distance from the ground truth (HigherHRNet-W48, 4.068 (±0.088); U-net [BGG16], 4.218 (±0.099); false-discovery-rate (FDR) adjusted *p* value < 0.01) (HigherHRNet-W48, 4.068 (±0.088); Baseline 101, 5.133 (±0.103); FDR adjusted *p* value < 0.01). Furthermore, Figure 7 demonstrates that the distance calculated using this model was significantly smaller than the distance based on evaluations by the residents. These findings indicate that the HigherHRNet-W48 model demonstrated superior accuracy compared to the residents.

### 3.2. Step 2: Inter-Rater Reliability and Intra-Rater Reliability in Qualified Images

**Figure 8:**
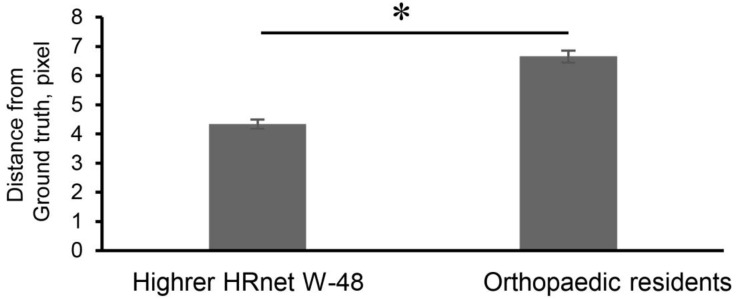
Comparison of distance from the ground truth of the apex points. * *p*  <  0.01 (Student’s *t*-test).

**Figure 9:**
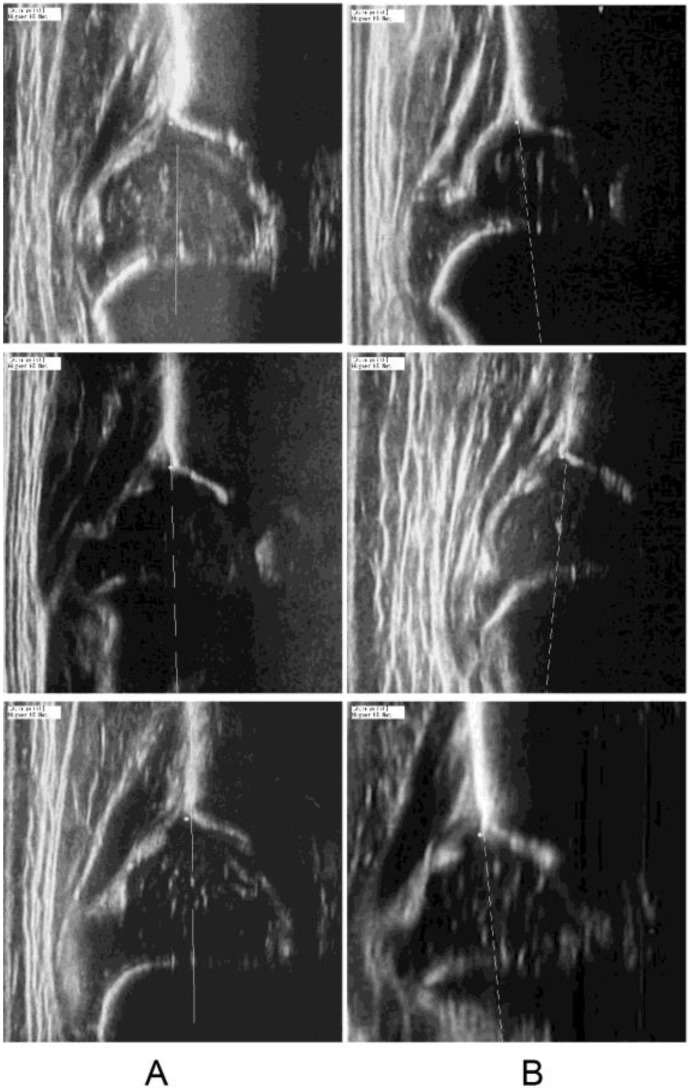
Examples of quality assessment. (**A**) Qualified images which have blurred bone contour of ilium. (**B**) Disqualified images in which the ilium was rotated.

**Figure 10:**
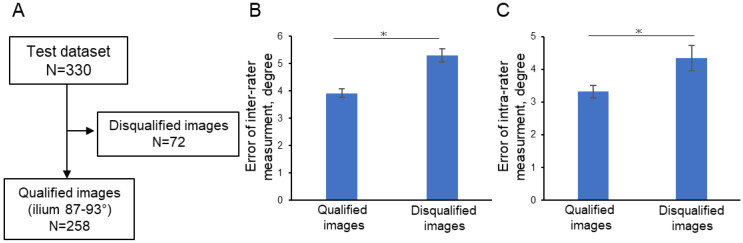
Depiction of quality assessment and validation: (**A**) Flow chart for quality assessment; (**B**) Error in inter-rater measurements of alpha angle, as evaluated by an orthopedic specialist; (**C**) Error in intra-rater measurements of alpha angle, as evaluated by an orthopedic specialist. * *p*  <  0.05 (Student’s *t*-test).

Unbiased quality assessments were conducted to evaluate the ultrasound images. Figure 8 provides examples of the quality assessments, showcasing both qualified images, which may contain blurred bone contours of the ilium, and disqualified images, in which the ilium appears rotated. The images were automatically divided into two categories: those that were qualified for use in diagnosis and those that were disqualified (Figure 9). This categorization is illustrated in Figure 10A. Next, the inter-rater reliability and intra-rater reliability for each image were analyzed and compared between these two groups. Figure 10B,C reveal that the qualified images had significantly lower error rates in both inter-rater and intra-rater measurements compared to the disqualified images. These findings emphasize the importance of rigorous quality control in the image selection process to ensure accurate diagnosis.

### 3.3. Step 3: Accuracy of Detecting DDH in Qualified Images

**Figure 11:**
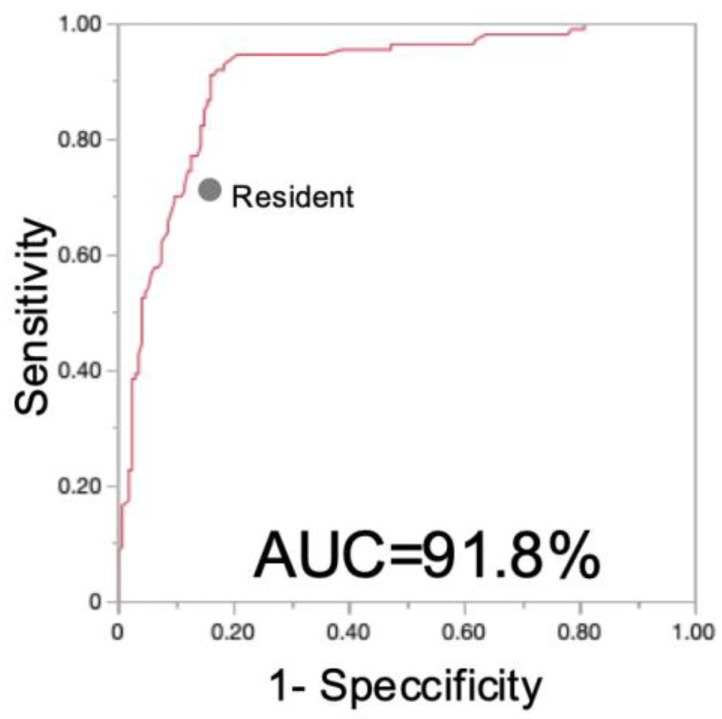
Receiver operating characteristics of our model for DDH diagnosis, juxtaposed with results from the orthopedic specialist. The system achieved an area under the curve (AUC) of 0.918.

**Figure 12:**
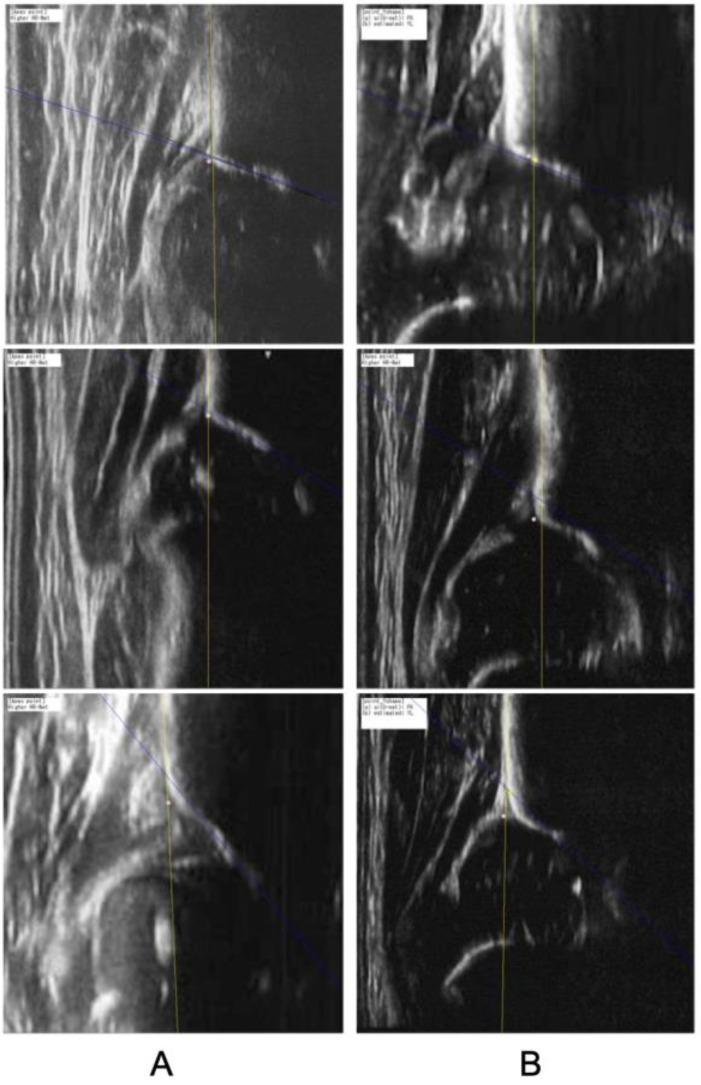
Examples of the generation of alpha angle. (**A**) Representative images (**B**) images with false analysis; discrepancy in estimated apex point (top); discrepancy in acetabular roof (middle and bottom).

Within the qualified images, the accuracy of DDH was visualized and is presented in Figure 11. The AUC based on the model was 0.918, indicating that the performance of the orthopedic resident trended below the model’s ROC curve. This comparison highlights the model’s enhanced ability to detect DDH, offering a potentially valuable tool for aiding clinicians in the diagnosis and early intervention of this common musculoskeletal abnormality in infants. The representative images and images with false analysis are shown in Figure 12.

## 4. Discussion

This study presented a deep learning-based model that efficiently evaluates infantile US images for DDH. The model’s unique approach, combining CNNs with traditional image processing, enables unbiased quality assessments of US images and automates the rendering of image quality. This is particularly relevant in clinical scenarios where consistent and objective evaluation is crucial. Moreover, the model’s ability to successfully navigate challenges such as rotated ilium and noise around the bone highlights its potential as a valuable tool for enhancing the screening and diagnosis of DDH in infants.

Our research introduced a fully automated system for detecting DDH with unbiased quality assessments, focusing on key challenges such as interference with similar tissues, reverberation noise, and rotation of subjects. The study utilized the concept of signal heterogeneity, commonly used in MRI for tumor characterization [33,34,35], applying it to US images of the bone region. Previous approaches based on contour shape detection failed due to issues like reverberation around the bone, leading to considerations of noise elimination. The study built upon previous work that showed that 3D technology could enable reliable analyses [17,18] and demonstrated that the bone region could be precisely evaluated by analyzing signal heterogeneity. The findings suggest that future research could focus on automatic noise reduction in US investigations on bone.

The diagnostic accuracy of our system was comparable to previous reports [6,8]. While existing studies on ultrasound images have primarily concentrated on segmenting essential structures such as the ilium [6,11], they have often overlooked the inherent challenges present in clinical settings. Artifacts, most notably the reverberations around bone, can be obstructive to making a correct diagnosis of DDH on ultrasound images in practice [13,14,15,16]. Thus, the previous models could face difficulties in analyzing images with artifacts since they do not include an algorithm for noise or artifact reduction. In fact, quality assessments are required for analyzing ultrasound images: interference with similar tissues, noise, such as reverberation, and the rotation of the subjects [12]. This study showed the comparative diagnostic value with the algorithms for noise reduction, excluding disqualified images, consistent with the standard protocols of DDH ultrasound examination.

In the specific approach employed in our proposed model, the initial step was to estimate the apex point, the key landmark. In the next step, bone regions (ilium and acetabular roof) were annotated by using image processing. The previous literature firstly segmented or detected the bone area, subsequently pointing out the key landmarks [6,11]. According to practical experience, and the corresponding evidence suggesting that artifacts around bone, such as reverberation, might affect the bone, our model changed the order of the steps. As a result, the initial step excluded the mimicking soft tissue of the ilium. Concerning the mean distance between the ground truth and the estimated apex points by HigherHRNet-W48, the diagnostic performance could have been improved if there had been more accurate detection models for estimating the apex model. Further studies could be investigated to refine the initial step.

The second step, which involved analyzing the bone area using the noise reduction algorithm, employed qualifying images according to the clinical protocols, where it is noted that the ilium should be vertical on the images. However, there have been no reports of strict criteria for the acceptable range of the iliac angle. In this clinical situation, we set it at 87–93° in this study. Using qualified images based on the eligible criteria, the diagnostic performance was comparative to the model of previous studies [6,11]. It should be considered that more strict criteria (e.g., set at 89–91) clearly reflect the definition of DDH, indicating the possibility that images with ilium at 87–89° or 92–93° were uncertain of DDH or not based on a strict protocol application.

The proposed model also had a unique feature, namely, the utilization of US images taken by non-specialist individuals, including orthopedic residents and those not specialized in ultrasonic investigation or pediatric orthopedics. Our dataset even contained images that were disqualified by traditional assessment, highlighting the potential of the system to assist novice sonographers. The ability of the system to help select a standard plane and make accurate diagnoses suggests that it could be a valuable tool for enhancing diagnostic accuracy, particularly for those new to sonography.

The deep learning-based models on medical images are roughly grouped into computer-aided diagnosis models and full-automatic diagnosis models; this model was consistent with the latter one. It is certainly true that orthopedics can improve the efficacy of DDH diagnosis by using computer-aided algorithms [36]. This can mean that orthopedics are able to make the correct diagnosis by combining those models. Still, given its prevalence as one of the most common congenital musculoskeletal abnormalities affecting infants [5], reliable screening tools to non-experienced orthopedics or novice sonographers are also required. They face difficulties even in relation to whether these images are qualified for the diagnosis, and then in how to determine the key structure and measure the critical angles. These issues would more likely be resolved by using fully automated models than computer-aided algorithms. Further prospective investigations should be performed to validate the performance of our proposed model by novice sonographers.

The limitations of our study warrant careful consideration. First, this system cannot evaluate the dynamic (movement) aspect of DDH screening, which was equally important as the static aspects and angle measurements. Second, the apex point was annotated by two pediatric orthopedic specialists. The subjectivity of these specialists and potential inter-rater variability could introduce bias into the dataset. Third, since there have been no reports of strict criteria for the acceptable range of the iliac angle, we set it at 87–93° in this study. Fourth, as the study was conducted within a single facility, the findings may reflect institutional practices and might not be universally applicable. To enhance the robustness and generalizability of our findings, future studies should ideally be designed as prospective endeavors, incorporating collaboration across multiple institutions. Such an approach would provide a more diverse and representative dataset, offering a more comprehensive understanding of DDH detection. Fifth, the dataset consisted of 1110 images from 417 infants, whereas some images were excluded manually. Some infants had twice the amount of acquisitions of ultrasound examinations or more. This unbalanced dataset, with the manual exclusion of images, could have affected the results. Sixth, we divided the images, not the infant, randomly into training and test dataset. There might have been data leakage. To minimize information leaks between the two datasets, only one image per joint was collected by the same examiner. Seventh, the diagnostic performance was not compared between several deep learning models, while the distance between the ground truth and the estimated one was shortest in HigherHRNet-W48. Eighth, the diagnostic performance was not evaluated by using N-fold cross validation. This procedure could have reduced the bias of the unbalanced dataset.

## 5. Conclusions

This study developed a hybrid machine learning model for the automated detection of DDH in infants with an AUC of 0.918, integrating 3D technology and deep learning to overcome challenges such as noise, rotation, and interference with similar tissues in ultrasound images. The study’s innovative approach, by analyzing signal inconsistency in 2D ultrasound images, demonstrated comparable accuracy to specialized orthopedic evaluations, even when using images taken by non-specialist individuals. Notably, the model’s capacity to evaluate images often disqualified by traditional assessment emphasizes its potential to assist novice sonographers and enhance diagnostic precision. The proposed model has the potential to ensure improved diagnostic performance in widely used 2D ultrasound examinations, especially by novice sonographers.

## Figures and Tables

**Table 1:** Demographic data.

	Training and Test Dataset
Number of infants	417
Median age, month	2
Female, %	81.7 (341/417)
Breech presentation, %	33.6 (140/417)
Family history of first relatives, %	20.6 (86/417)
Skin laterality, %	43.2 (180/417)
Limb limitation, %	25.71(105/417)
Unstable hip, %	22.1 (92/417)

**Table 2:** Comparison of convolutional network model for estimating the apex point.

Name	Mean Distance	SE	Params (M)	FLOPs (G)	Duration (ms)
HRNet-W32	4.168	0.096	28.54	10.27	26.187
HRNet-W48	4.120	0.091	63.59	21.02	27.297
HigherHRNet-W32	4.149	0.101	28.63	11.78	25.32
HigherHRNet-W48	4.068	0.088	63.8	24.39	25.655
Baseline 50	9.319	0.165	34	12.9	7.586
Baseline 101	5.133	0.103	52.99	17.77	9.218
Baseline 152	5.171	0.107	68.63	22.64	13.232
U-net (VGG11)	4.419	0.103	9.28	9.82	1.439
U-net (VGG13)	4.457	0.108	9.46	14.68	1.984
U-net (VGG16)	4.218	0.099	14.77	20.12	2.271
U-net (VGG19)	4.462	0.113	20.09	25.57	2.688
U-net (MobileNetV2)	4.890	0.096	6.63	3.38	9.132
U-net (MobileNetV3 large)	4.507	0.095	6.69	3.08	8.092
U-net (MobileNetV3 small)	7.136	0.225	3.59	2.56	6.614

Mean distance: mean distance from ground truth and estimated apex point (pixel), SE: standard error, FLOPs: floating point operations per second.

## Data Availability

The data presented in this study are available upon request from the corresponding author.

## Abbreviations

DDH	Developmental Dysplasia of the Hip
CNN	convolutional neural network
ROI	region of interest
eAP	estimated apex points
ROC	receiver operating characteristic
